# Burnout and work-life balance: the generational points of view

**DOI:** 10.1186/s13244-026-02232-5

**Published:** 2026-03-02

**Authors:** Isabel Molwitz, Amine Mohamed Korchi, Ioana Andreea Gheonea, Luis Curvo-Semedo, Gennaro D’Anna

**Affiliations:** 1 Department of Diagnostic and Interventional Radiology and Nuclear Medicine, University Medical Center Hamburg-Eppendorf, Hamburg, Germany; 2 Imaging Center Onex, Groupe 3R, Geneva, Switzerland; 3 Singularity Consulting, Geneva, Switzerland; 4 Department of Radiology and Medical Imaging, University of Medicine and Pharmacy, Craiova, Romania; 5 Department of Imaging, Local Health Unit—Aveiro Unit, Aveiro, Portugal; 6 Faculty of Medicine, University of Coimbra, Aveiro, Portugal; 7 Department of Medical Sciences, University of Aveiro, Aveiro, Portugal; 8 Department of Diagnostic Imaging and Stereotactic Radiosurgery, Centro Diagnostico Italiano S.p.A, Milan, Italy

**Keywords:** Work-life balance, Generational differences, Radiologist burnout, Healthcare workforce, Intergenerational collaboration

## Abstract

**Abstract:**

Work-life balance has emerged as a central theme in modern medicine, particularly in radiology, where high burnout rates underscore the urgency for systemic change. This narrative review explores how perceptions of work-life balance vary across generations—Baby Boomers, Generation X, Millennials, and Generation Z—and how these differences shape workplace expectations and cultural evolution within healthcare. Baby Boomers often view medicine as a vocation requiring sacrifice and long hours, while Gen X emphasises flexibility and independence. Millennials prioritise purpose, inclusivity, and work-life integration, favouring fluid schedules and value-driven environments. Gen Z, as digital natives, seeks ethical workplaces, diversity, and clearly defined personal-professional boundaries.

That paper started from a dedicated session at the European Congress of Radiology (ECR) 2025, combining literature references with reflections on evolving professional values. It highlights that while generational perspectives differ, common ground exists: across all groups, well-being, fulfilment, and supportive workplace structures are increasingly seen as essential rather than optional. The paper emphasises the importance of adapting institutional policies to accommodate generational needs through flexible scheduling, mentorship, protected time, and inclusive leadership.

Ultimately, we aim for the embracing of intergenerational collaboration and recognition of the diverse definitions of professional success, which are key to building resilient radiology teams. Sustainable solutions must move beyond one-size-fits-all models to foster innovation, prevent burnout, and retain talent across all career stages. It is also calling for healthcare institutions to proactively integrate these perspectives to shape a more supportive and effective professional culture.

**Critical relevance statement:**

This paper offers a narrative overview of generational perspectives on work-life balance in radiology, highlighting both shared values and evolving priorities across age groups

**Key Points:**

- Burnout remains a widespread issue in radiology, with high prevalence across all career stages, emphasising the need for systemic solutions rather than individual resilience alone.
- Generational views on work-life balance vary: Boomers value duty, Millennials seek purpose and flexibility, while Gen Z demands ethics, diversity, and personal sustainability.
- Intergenerational collaboration and adaptability are essential for building resilient teams, requiring healthcare institutions to embrace diverse expectations and implement inclusive, flexible work models.

**Graphical Abstract:**

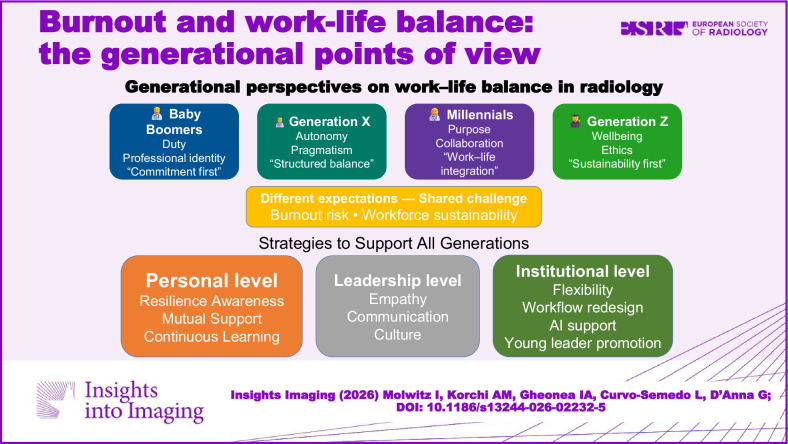

## Introduction

The concept of work-life balance has evolved into a central tenet of modern professional life, influencing how individuals assess job satisfaction, performance, and long-term career planning. In the field of medicine—and radiology in particular—this theme is especially salient given the demanding nature of the profession. These demands include long hours, high responsibility, and constant exposure to occupational stress with long days in obscurity, repetitive tasks, visual stress, a high level of pixel/frames visualisation, and organisational change that intersect with the need for personal well-being [1]. Indeed, burnout syndromes have been found to be prevalent in more than 50% of radiologists and already occur among radiological trainees. Radiology is one of the medical specialities with the highest burnout rates [2–4].

Work-life balance is not a static or universal concept. It is deeply shaped by generational values, socio-economic factors, and evolving cultural expectations. What was once considered a privilege is now often regarded as a right, especially among younger professionals [5]. However, it is not only an individual concept but has a high economic impact, as well. Physician burnout imposes significant financial burdens on healthcare organisations, with costs ranging from $500,000 to over $1 million per affected physician due to recruitment, lost billings, and onboarding expenses of replacing employees [6]. Beyond these direct costs, burnout leads to indirect consequences such as increased medical errors, higher malpractice risks, reduced patient satisfaction, and damage to organisational reputation [7]. On the contrary, a balanced work-life dynamic enhances job performance [8–10] and organisational commitment [11, 12] and thus loyalty towards one employer. Also, work-life balance increases job satisfaction [8, 13, 14] and career development [8, 15] while mitigating anxiety, depression, stress [16], and burnout [11, 17].

The differing perceptions of work-life balance across generations—ranging from Baby Boomers to Gen Z—reveal not only contrasting priorities but also opportunities for dialogue and structural improvement [18]. We discussed these different points of view in a session of the European Congress of Radiology (ECR) in 2025. This narrative review aims to describe the concluded generational perspectives on work-life balance in healthcare, with a focus on radiologists. By comparing expectations, experiences, and values across age groups, we aim to understand how each cohort interprets professional fulfilment and personal sustainability, and how healthcare institutions can adapt to build resilient, multigenerational teams.

## The different generations: a definition

In this manuscript, we use “generation” in the social-science sense: a birth-year cohort whose members share a location in historical time and formative experiences that shape attitudes, norms, and work behaviours [19]. Because there is no single official standard for generational cut-points, boundaries can vary across sources [20]. For analytic coherence with the rest of our paper (training eras, digital nativity, expectations around flexibility and leadership), we adopt a pragmatic scheme drawn from prominent references: Baby Boomers (1946–1964), Generation X (1965–1980), Millennials (1981–1996), and Generation Z (1997–2012), with Generation Alpha (2013+) where needed (Table 1). These ranges, especially the Millennial/Gen Z boundary at 1996/1997, are used here as analytic tools, not fixed identities, allowing us to interpret radiology-specific patterns in work–life balance across cohorts while acknowledging edge-year overlap and within-group heterogeneity [21].

**Table 1:** Events and values of the generations are evaluated

Values are influenced by historical events			
EVENTS			
Baby Boomers (1946–1964)	Gen X (1965–1980)	Millennials/Gen Y (1981–1996)	Gen Z (1997–2012)
Post-war reconstruction Cold War and nuclear threat Social reforms and the welfare state European integration	End of the Cold War Economic crises and free-market reforms Digital revolution Cultural pluralism	9/11 and terrorism 2008 financial crisis Internet and social media Rising climate awareness	Smartphone ubiquity & platform ecosystems Pandemic schooling (COVID-19) Creator economy Climate activism Renewed geopolitical instability

VALUES			
Baby boomers	Gen X	Millennials/Gen Y	Gen Z
Work ethic and loyalty Optimism and idealism Preference for traditional stability Clear hierarchies	Adaptability and resilience Independence and pragmatism Early focus on work–life balance Healthy scepticism toward institutions	Mobile-first adopters Collaboration and inclusivity Purpose and flexibility Mentorship and continuous feedback	Digital natives Psychological safety and mental-health awareness Values-aligned employers Structured feedback Financial planning/security

In our framework, historical events act as formative anchors that shape cohort-level value patterns relevant to work–life balance and team dynamics. Baby Boomers (1946–1964) came of age amid post-war reconstruction, the Cold War and the rise of the welfare state, fostering a duty-first work ethic, loyalty to institutions, and a preference for clear roles and traditional stability—traits that map to comfort with in-person collaboration and hierarchical decision pathways in clinical services. Generation X (1965–1980) entered the workforce around the end of the Cold War and early digitalisation, through economic turbulence and market liberalisation; these contexts reinforced adaptability, pragmatism, and independence, with an early, explicit attention to work–life balance and outcome-based performance—often translating into acceptance of hybrid work and autonomy in scheduling. Millennials/Gen Y (1981–1996) were shaped by 9/11, the 2008 financial crisis, and the rise of the social web; they tend to value collaboration and inclusivity, seek purpose and flexibility, and appreciate mentorship and continuous feedback—aligning well with multidisciplinary practice, agile rotas, and supportive wellness policies. Generation Z (1997–2012), as true digital natives, experienced smartphone ubiquity, pandemic schooling, and climate activism; they prioritise psychological safety and mental-health awareness, values-aligned employers, structured feedback, and financial security—preferences that resonate with transparent leadership, clear progression criteria, and AI-enabled, team-based workflows.

These tendencies are analytic heuristics, not prescriptions: individual variation is wide, and overlap at cohort edges is expected, but the events–values link offers a practical lens to tailor communication, scheduling, and leadership across a multigenerational radiology workforce.

### The baby boomers' point of view

The concept of work-life balance has evolved significantly across generations in medicine, particularly within the field of radiology. For Baby Boomers (born 1946–1964), medicine was often seen as a calling, or a sort of “vocation” that demanded dedication, long hours, and personal sacrifice

Baby boomers in radiology are often characterised by a strong commitment to their profession, with a tendency to equate long hours and extra responsibilities with dedication and engagement. Many baby boomers may view requests for flexible hours or reduced workloads as a sign of disengagement or a lack of willingness to contribute fully, reflecting the traditional expectation of sacrificing personal time for professional advancement [22].

However, there is increasing recognition among some baby boomers of the importance of flexibility, particularly as more trainees balance family life and work. Leaders from this generation acknowledge the need to support younger colleagues who are navigating parenthood during their training, a shift from past norms. Baby boomers also value professionalism, social interaction in the workplace, and staff diversity, which contribute to their overall job satisfaction [3].

While baby boomers differ in their baseline attitudes, there is respect for clinical excellence and a shared recognition of radiology’s cognitive and diagnostic complexity, as in all generations. Tensions can arise, however, in academic or hospital settings where Baby Boomers may see newer expectations for work-life balance as incompatible with traditional standards of dedication or coverage.

Yet many practices have found ways to bridge this generational divide by fostering team-based models, implementing wellness initiatives and recognising diverse definitions of professional fulfilment.

Baby Boomers have increasingly come to appreciate the importance of sustainability in medical careers.

Their perspectives continue to shape the culture of radiology, informing how departments organise work, support wellbeing, and prepare for the next generation of physicians [23, 24]. The potential of baby boomers to support the following generations and shape the work environment to ensure employee well-being is thus enormous.

Baby Boomers often view burnout as a challenge to be endured rather than openly discussed. Shaped by post-war values of loyalty and work ethic, they tend to stay committed despite stress, which can delay recognition of burnout symptoms. Preventive strategies are often informal and based on personal resilience or routine. Seeking mental health support may still carry stigma for this group, although that is slowly changing.

## The Gen X point of view

Generation X (born 1965–1980), came of age during an era of increasing specialisation, technological advancement, and shifting social values, which reshaped their views on how career and personal life should coexist [22]. Generation X radiologists, shaped by societal changes such as increased divorce rates and less stable households, prioritise work-life balance and are less willing to compromise family time for additional work responsibilities. Gen Xers tend to value independence, efficiency, and results over traditional processes. They advocate for predictable work hours, the ability to work remotely, and flexible scheduling to accommodate family and personal commitments [25]. This generation sees technology as a tool to facilitate work outside the traditional office, and they are comfortable with nontraditional work arrangements, such as working from home or outside standard hours. Gen Xers are also more likely to seek real-time feedback, prefer flatter organisational hierarchies, and believe that merit should be the basis for respect and advancement rather than seniority [22].

Job satisfaction for Gen X radiologists is closely tied to job security, compensation, and workplace flexibility [26]. Gen X radiologists acknowledge the foundation of excellence laid by their predecessors but may perceive an inflexible culture as resistant to necessary change.

### Millennials and the new work-life balance: a paradigm shift

The millennial generation, born between 1980 and 1995, has emerged as the largest and most influential workforce of our time. This group, highly educated, technologically adept, and culturally diverse, has reshaped professional landscapes, setting new expectations for career growth, workplace culture, and the integration of work with personal life.

Millennials prioritise purpose and well-being over pay. Their career trajectories are marked by frequent moves, non-linear progressions, and an inclination toward freelancing and entrepreneurship. Unlike previous generations, who sought stability and long-term employment, millennials seek opportunities that align with their values and offer continuous growth.

Traditional corporate hierarchies are being replaced with flatter, more collaborative structures. Millennials thrive in environments that encourage open communication, mentorship, and real-time feedback rather than rigid annual performance reviews. They seek workplaces that prioritise mental health, inclusivity, and transparency, rejecting toxic cultures in favour of those that foster well-being.

A defining trait of this generation is its digital-first mindset. Millennials are natural adopters of technology, leveraging digital tools to enhance productivity and maintain a seamless integration between work and life. They expect workplace technology to mirror personal tech experiences—intuitive, efficient, and adaptable [27].

For millennials, work is not a place you go to; it is something you do [28]. The traditional boundaries between work and personal life have dissolved in favour of a more fluid approach, where flexible schedules and remote work enable effectiveness without sacrificing personal time. They prioritise efficiency, using automation and productivity tools to streamline tasks while maintaining autonomy. This shift reflects the broader phenomenon of work-life blending, in which professional and private domains increasingly overlap and influence one another, creating flexible but more permeable boundaries [29].

Rather than subscribing to the rigid work-life balance model, which implies a strict separation between professional and personal spheres, millennials champion work-life integration. They seek roles that allow them to align their careers with their personal aspirations, ensuring fulfilment in both domains.

Millennials have navigated economic recessions, global crises, and rapid technological disruptions, making adaptability and resilience their defining traits. In the healthcare sector, for instance, rising demands, declining reimbursements, and the high-burnout rates exceeding 50% among radiologists [30] pose significant challenges [22]. To address these issues, millennials emphasise leadership, operational excellence, and the strategic use of AI and technology to optimise efficiency while maintaining well-being.

Moreover, this generation places a strong emphasis on social responsibility. They are drawn to organisations that advocate for sustainability, equity, and inclusivity. Companies that align with these values are more likely to attract and retain millennial talent [31].

The key to a thriving workplace culture lies in flexibility, inclusivity, and mentorship. Purpose-driven organisations that recognise and cultivate these values will not only attract top talent but also build sustainable, multi-generational and high-performing teams capable of shaping the future of healthcare and beyond [23].

Millennials are more vocal about burnout and proactive in addressing it. Having grown up with digital access and open discussions around mental health, they are likely to seek help, use wellbeing resources, or even change careers if a job feels unsustainable. They emphasise meaningful work, psychological support, and flexible, purpose-driven environments as key to prevention, and expect institutions to take responsibility.

Summarising, Millennials are not just redefining work; they are reshaping industries. Their approach to career development, workplace culture, and work-life integration is setting new standards that organisations must adapt to in order to remain competitive. In healthcare, radiology, and countless other fields, the lessons from millennials are clear: embrace change, prioritise purpose, and leverage technology to create a fulfilling and efficient work environment. As we move forward, the focus should not be on resisting these changes but on learning from them—after all, the future of work belongs to those who can adapt and thrive in this new paradigm [31].

### Work-life-balance—the Gen Z point of view

Generation Z is known for its emphasis on work-life balance. It includes individuals born between the mid-1990s and early 2010s [32]. Members of Generation Z are the first to grow up in a completely digital age [33] and are thus assumed to be digital natives. Further characteristics associated with Generation Z include prioritising diversity and inclusivity [33]. Besides work-life balance, they search for meaningful jobs [33] with high ethical and moral standards [34].

In the discussion of work-life balance expectations of Gen Z, it is frequently assumed that members of Gen Z are less willing to work as intensively as other generations. However, this is not supported by evidence. A study involving 1790 high-school students interested in medical careers revealed that a significant proportion of Gen Z values altruism, intrinsic motivation, and high performance [35]. Many within this group are career-oriented yet strongly emphasise maintaining a fulfilling personal life alongside professional ambitions. Work-life balance is simply perceived as a critical factor influencing their job choices. Also, they are not alone in this. A study among 510 radiologists of all age groups working in Germany revealed that all participants judged joy at work and a good work atmosphere as their most important expectation towards their professional life [36]. The second most important criteria for all age groups, except for chief physicians, were reliable work times, planning security, and family friendliness. Interestingly, within this study, residents and specialists representing the younger generations judged a good income as less relevant than the expectations mentioned above. In contrast, a good income was more critical for radiologists in private practices and chief physicians who are typically more senior. The changing impact of financial remuneration, especially when in exchange for an insufficient work-life balance, is a well-known characteristic of Generation Z. The same study showed discrepancies between expectations and reality [36]. Reliable work time, planning security, and family friendliness were not considered sufficiently fulfilled, except for radiologists working in outpatient care.

One aspect that might have influenced the relevance of work-life balance among the younger generations is the increasing workload and more demanding economic restraints in medicine and radiology. In radiology, an enormous and ongoing growth of relative value units has been documented from the early 90 s onwards [37, 38]. Generation Z members are thus objectively at a higher risk for burnout. Being aware of this risk and experiencing the high-throughput work environment, they prioritise their health over the system’s monetary output.

To integrate Generation Z successfully, organisations must thus implement flexible work hours, remote work opportunities, reliable schedules, protected research time, and structured training programs to fulfil expectations on work-life balance. These measures would benefit not only Gen Z but also the well-being of all generations. Moreover, they can be of economic value, as companies that foster such a work environment can experience enhanced employee engagement, lower turnover rates, and improved overall productivity. Ultimately, acknowledging without prejudice and adapting to the evolving work expectations of Gen Z is crucial for fostering a sustainable and resilient workforce in the future.

## Conclusions and actionable insights

Work-life balance has evolved from a personal aspiration to a collective imperative across generations in the medical profession. From the steadfast resilience of senior physicians shaped by sacrifice and duty to the transformative mindset of millennials who prioritise purpose, flexibility, and integration, and the value-driven, technologically native Gen Z demanding ethical workplaces and personal sustainability, each generation brings distinct yet complementary visions of what it means to thrive professionally.

The workplace of the future must embrace intergenerational collaboration. Baby Boomers, Gen X, Millennials, and Gen Z bring diverse but complementary perspectives that, when leveraged effectively, can drive innovation and workplace transformation. Success is rooted not in rigid policies but in open communication, empathy, and shared learning.

While generational differences can at times appear divisive, these reflections reveal common ground [26]: across all age groups, there is a growing recognition that well-being, fulfilment, and organisational support are not luxuries, but prerequisites for high-quality, sustainable healthcare [30]. Burnout, once seen as an individual weakness, is now rightly viewed as a systemic issue requiring structural change and cultural evolution.

To build resilient, multigenerational radiology teams, healthcare institutions must move beyond one-size-fits-all solutions [39]. Embracing flexible work models, fostering mentorship across generations, and promoting open communication are essential steps [40]. Only through mutual respect and the integration of diverse generational values can we ensure a work culture that not only prevents burnout but also attracts, retains, and uplifts talent—ultimately benefiting both clinicians and the patients they serve.

To address burnout among radiologists, a combination of individual, leadership, and organisational strategies is essential [41, 42].

We propose the following:

- At the personal level, we need to become aware of our individual limitations by promoting self-reflection, fostering resilience, and finding the courage to demand flexible work times or time off, e.g. for sabbaticals to restore our well-being. Mindfulness-based interventions [43] have demonstrated efficacy in reducing stress and enhancing focus among physicians [44]. Radiologists who participated in resilience workshops reported reduced burnout scores and improved coping strategies [45]. Accessing existing professional support programs can also enhance impact.
- Leadership plays a critical role; leaders should be empathetic and open to their team's well-being. By supporting team morale, they will gain excellence. Developing these leadership skills is a key preventive measure which would be mandatory before promotion into a leadership function.
- Institutions should shift focus from fixing individuals to improving workflows, offering structured wellness initiatives, and allowing flexible work models.
- The integration of AI and decision-support tools holds promise for reducing repetitive tasks and improving work satisfaction, though implementation must avoid introducing new stressors [46]. Moreover, institutional policies, such as incorporating well-being metrics into departmental performance evaluations, may embed wellness into organisational structures.

Ultimately, lasting solutions require both systemic change and a supportive culture, not just personal coping strategies.

In conclusion, despite generational differences, the passion for radiology joins all generations. And only in a joint effort of all generations, accepting the importance of personal well-being in order to best support our patients, can we thrive in future.
